# High expressions of CD10, FAP and GPR77 in CAFs are associated with chemoresistance and worse prognosis in gastric cancer

**DOI:** 10.3389/fonc.2022.984817

**Published:** 2022-10-28

**Authors:** Yilin Tong, Zehua Zhao, Jianjun Zhang, Wentao Wang, Yanmei Zhu

**Affiliations:** ^1^ Department of Gastric Surgery, Affiliated Cancer Hospital of Dalian University of Technology (Liaoning Cancer Hospital and Institute, Cancer Hospital of China Medical University), Shenyang, Liaoning, China; ^2^ Department of Pathology, Affiliated Cancer Hospital of Dalian University of Technology (Liaoning Cancer Hospital and Institute, Cancer Hospital of China Medical University), Shenyang, Liaoning, China

**Keywords:** gastric cancer, tumor regression grade (TRG), neoadjuvant therapy, cancer-associated fibroblasts (CAFs), CD10, FAP, GPR77

## Abstract

**Introduction:**

As neoadjuvant chemotherapy (NCT) has been successfully introduced in gastric cancer (GC), more biomarkers are needed to evaluate the efficacy. Cancer-associated fibroblasts (CAFs) is associated with chemoresistance and prognosis. Three biomarkers, CD10, fibroblast activation protein-α (FAP) and G-protein-coupled receptor 77 (GPR77), have been proved to express in CAFs. However, their predictive values for efficacy of NCT and prognosis in gastric cancer is unknown.

**Methods:**

Totally, specimens of 171 locally advanced gastric cancer patients who underwent NCT and D2 radical gastrectomy and matched preoperative biopsy specimens were retrospectively analyzed. Tumor regression grade (TRG) is reevaluated according to Mandard TRG. Expressions of CD10, FAP and GPR77 in CAFs before NCT (pre-) and after NCT (post-) were evaluated by immunohistochemistry. Survival curves on overall survival (OS) were obtained by Kaplan-Meier method, and differences were analyzed by log-rank test. Associations between categorical variables were explored by chi-square test or Fisher’s exact method. Univariable and multivariate analyses were performed by logistic regression model and Cox proportional hazard regression model.

**Results:**

High expressions of post-CD10, post-FAP, post-GPR77 and pre-CD10 were related to worse TRG (all p<0.05). In multivariable analysis, post- and pre-FAP were independent predictive factors to TRG (p<0.010). Post-CD10 (p=0.032) and post-FAP (p=0.013) were related to OS in univariable analysis, but none of biomarkers were independent prognostic factors in multivariable analysis.

**Conclusions:**

Expressions of CD10, FAP and GPR77 in CAFs were related to chemoresistance and overall survival, and these biomarkers have predictive values for tumor regression and prognosis in locally advanced gastric cancer patients.

## Introduction

As neoadjuvant chemotherapy (NCT) followed by surgery has become a recommended treatment plan for locally advanced gastric cancer (GC) (1), the evaluation of the effectiveness of NCT is becoming increasingly important. Pathologically, TNM system (2) and tumor regression grade (TRG) (3) were widely used to evaluate the efficacy of NCT qualitatively and quantitatively. However, these two methods rely heavily on the subjective judgment of pathologists, which means subjective bias is inevitable. Therefore, more objective evaluation indicators are needed. On this point, the expressions of biomarkers are promising.

Cancer-associated fibroblasts (CAFs), one of the primary stromal cell types in the tumor stroma, play an important role in chemoresistance and tumor progression (4), by secreting cytokines, chemokines and exosomes (5, 6), remodeling the extracellular matrix (ECM) (7), facilitating angiogenesis, suppressing antitumor immune responses, and promoting resistance to therapy (8). Many biomarkers have been used to identify CAFs, and different biomarkers showed various functions.

Fibroblast-activation protein (FAP), a type II integral membrane protein that belongs to the membrane-bound serine protease family, has been used as a specific marker of activated CAFs (9). FAP could promote cell proliferation and migration by various processes, such as producing ECM (10), increasing growth factors (11) facilitating angiogenesis (12) and regulating antitumor immune response (13). FAP could also regulate drug sensitivity by interacting with membrane proteins (14), or increasing the expression of chemokines (15). FAP showed a prognostic value in many cancers, and a high expression of FAP tended to predict a poor prognosis (16–18). However, whether FAP has predictive values on efficacy of NCT and prognosis in locally advanced GC patients who underwent NCT is still unknown.

CD10, a cell surface zinc-dependent metalloprotease that could regulate the biological activities of various peptide substrates (19), has been proved to be correlated with tumor progression and aggressiveness in many cancers such as melanoma (20), colorectal cancer (21) and breast cancer (22). G-protein-coupled receptor 77 (GPR77, also named C5aR2 and C5L2), one of the C5a receptors, is a powerful regulator of immune function. Activation of GPR77 could regulate cytokine production including IL-6 and TNF-α, which are important for tumor promotion and progression (23). CD10 and GPR77 have been recently proved to express on a CAFs subset, and correlate with cancer formation, chemoresistance and poor survival in breast and lung cancer patients (8). However, the expression of these two biomarkers in CAFs in gastric cancer and their predictive values are still unknown.

In this study, we assessed the expressions of FAP, CD10 and GPR77 in CAFs of gastric cancer samples after and before treatment, and evaluated their predictive values on the efficacy of NCT and prognosis in locally advanced gastric cancer patients.

## Material and methods

### Patients

All patients with gastric cancer between January 2010 and June 2018 at our institute were reviewed. Patients fulfilled the following inclusion criteria were included (1): pathologically confirmed gastric adenocarcinoma; (2) locally advanced gastric cancer (8th American Joint Committee on Cancer [AJCC] clinical stage: cT2N1M0-T4N3M0, II-III); (3) underwent NCT with or without postoperative therapy; (4) received curative gastrectomy surgery; and (5) specimens before and after treatment were available. Patients with following exclusion criteria were excluded: (1) underwent preoperative radiotherapy; (2) suffering from gastric remnant cancer or other malignant tumors; (3) incomplete information on staging or therapy; or (4) insufficient slices or blocks to evaluate biomarkers. After selected, 171 patients met the inclusion criteria of our study. For NCT therapy, 133 (77.8%) patients underwent SOX, 28 (16.4%) patients underwent FOLFOX and 10 (5.8%) patients underwent XELOX.

### Pathological response assessment

All slides and blocks indicating surgical specimens (post-treatment) and diagnostic specimens (pre-treatment) were retrieved from the biospecimen library of our hospital and were separately reviewed by two experienced gastrointestinal pathologists (Y.Z. and D.L.). TNM stage was reevaluated according to the eighth edition of the AJCC cancer staging guideline. Histological regression grade of the primary tumor was assessed according to the Mandard system: TRG 1 (complete fibrosis with no evidence of residual tumor, i.e., complete regression), TRG 2 (fibrosis with rare tumor cells), TRG 3 (fibrosis and residual tumor with a dominance of fibrosis), TRG 4 (fibrosis and residual tumor with a dominance of tumor), and TRG 5 (extensive residual tumor without evidence of regression). When disagreement appeared between pathologists, an agreement would be reached by joint rereview and discussion through a multihead microscope. Other extracted histopathological characteristics were reconfirmed during the evaluation process.

### Immunohistochemical staining

CAFs biomarkers including CD10, FAP and GPR77 were assessed by immunohistochemical staining method. Immunohistochemical staining method in this study was based on and modified from the method in Su et al. article (8). Briefly, specimens were incubated with specific primary antibodies (for CD10, a mouse monoclonal primary antibody (Ready-to-use; Maxim, Fujian, China) incubated at room temperature for an hour; for FAP, a rabbit monoclonal primary antibody (1:800 dilution; Boster, California, USA) and GPR77, a rabbit polyclonal primary antibody (1:400 dilution; Abcam, Shanghai, China): overnight at 4°C).

### Assessment of immunohistochemical staining

For every biomarker, the result of immunohistochemistry was evaluated by the total point, which was equal to the score of the proportion of positive area multiplied by the score of the intensity of the staining. The score of the proportion of positive area was defined as score 0-4, score 0: <1% positive area; score 1: 1%-25% positive area; score 2: 25%-49% positive area; score 3: 50%-74% positive area; and score 4: 75%-100% positive area. The intensity of the staining was defined as score 1-3: score 1: slight, light brown linear or granular staining on the cell membrane or cytoplasm; score 2: moderate, brown linear or granular staining on the cell membrane and cytoplasm; and score 3: strong, dark brown linear or granular staining on the cell membrane and cytoplasm. The total point was divided into 4 groups: 0: 0 point; 1+: 1-2 points; 2+: 3-4 points; and 3+: >4 points. For all biomarkers, the results of immunohistochemistry were divided into negative (total score 0 and 1+) and positive (total score 2+ and 3+). FAP was found to be expressed in cytoplasm of CAFs, while CD10 and GPR77 was found to be expressed in cytoplasm and cell membrane of CAFs.

### Statistical methods

Relationships among categorical variables were investigated by the chi-square test or Fisher’s exact test. Logistic regression analysis was used to explore the factors associated with pathological response. Cox regression analysis was used to assess the risk factors on overall survival (OS), and factors with p-value < 0.05 were included in the multivariable analysis. Because of collinearity between ypN and ypTNM, ypTNM was not included in multivariable analyses. Survival curves for OS were obtained using the Kaplan-Meier method, and the log-rank test was used to compare differences. All patients were followed up every 3 months during the first 2 years, every 6 months for the following 3 years and annually thereafter. OS was the time from initial treatment to death from any cause or last date of follow-up. Data were proceeded by SPSS ver. 25.0 (IBM Corp., Armonk, NY) and R 3.6.1 software (R Foundation for Statistical Computing, Vienna, Austria).

## Results

### Assessment of pathological response

**Figure 1:**
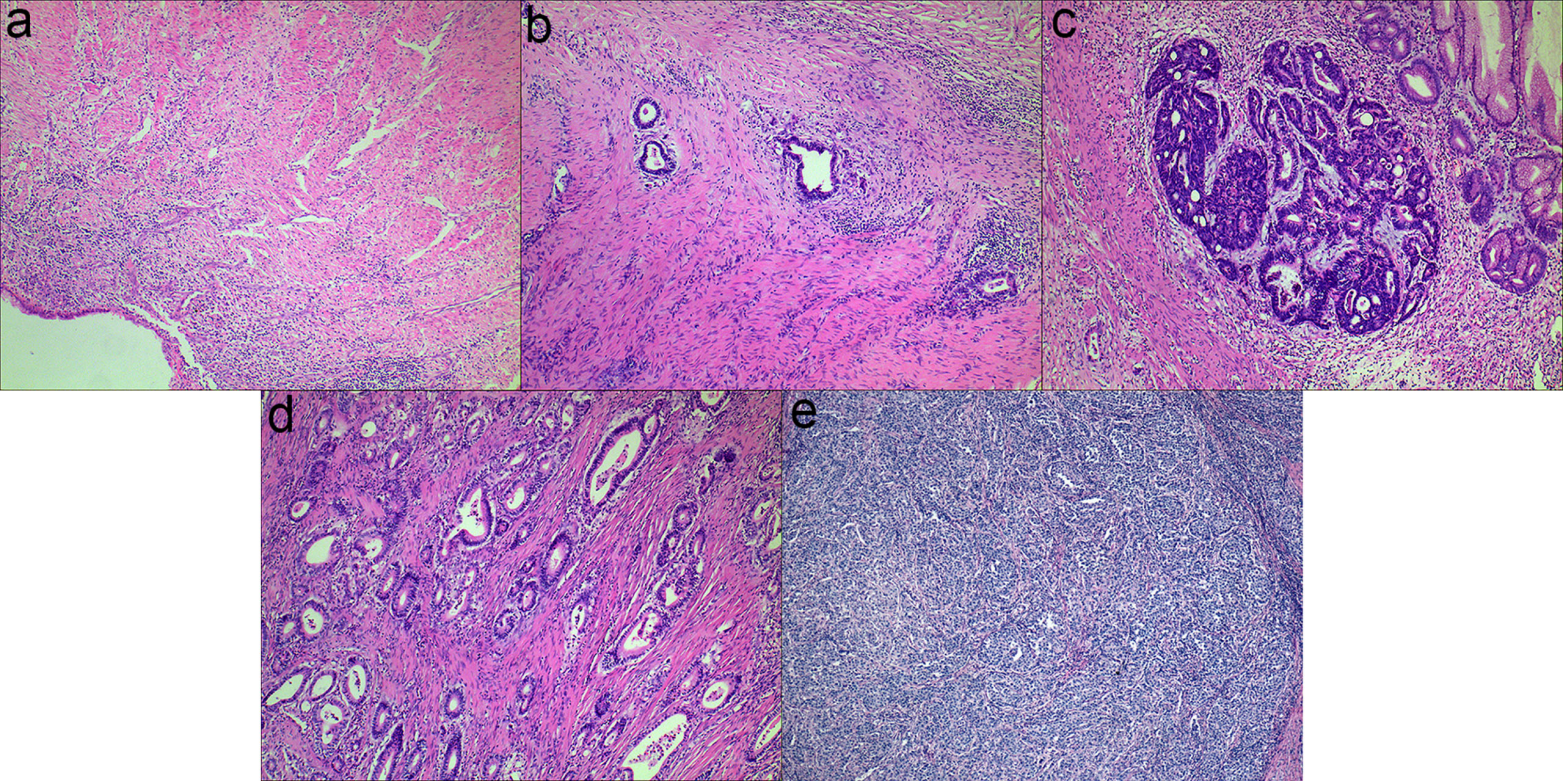
Examples of Mandard TRG (a-e): **(A)** TRG 1, complete tumor regression; **(B)** TRG 2, rare residual tumor; **(C)** TRG 3, more residual tumor but less than fibrosis; **(D)** TRG 4, residual tumor with signs of regression; **(E)** TRG 5, residual tumor without regression.

The examples of Mandard TRG are shown in **Figure 1**. Totally, 797 slides indicating surgical specimens were reviewed. The median number of reviewed slides was 4, with an interquartile range from 3 to 5. After revaluation, the number of patients was 10, 48, 57, 50 and 6 in the group of TRG 1-5, respectively. There was no significant difference in survival between TRG 1 and TRG 2 (p=0.374), so these two categories were classified into the responder group. Similarly, no significant difference was found among TRG 3, TRG 4 and TRG 5 (p=0.560), so these two categories were classified into the non-responder group. The survival curves of Mandard TRG were shown in **Supplementary Figure 1**.

### Expressions of biomarkers and correlations with pathological response

Morphologically, CAFs are a group of spindle-shaped cells surrounding the tumor cells. In 171 specimens after treatment, in CAFs, 31 cases were positive for CD10, with a positive rate of 18.1%; 68 cases were positive for FAP, with a positive rate of 39.8%; 21 cases were positive for GPR77, with a positive rate of 12.3%.

**Figure 2:**
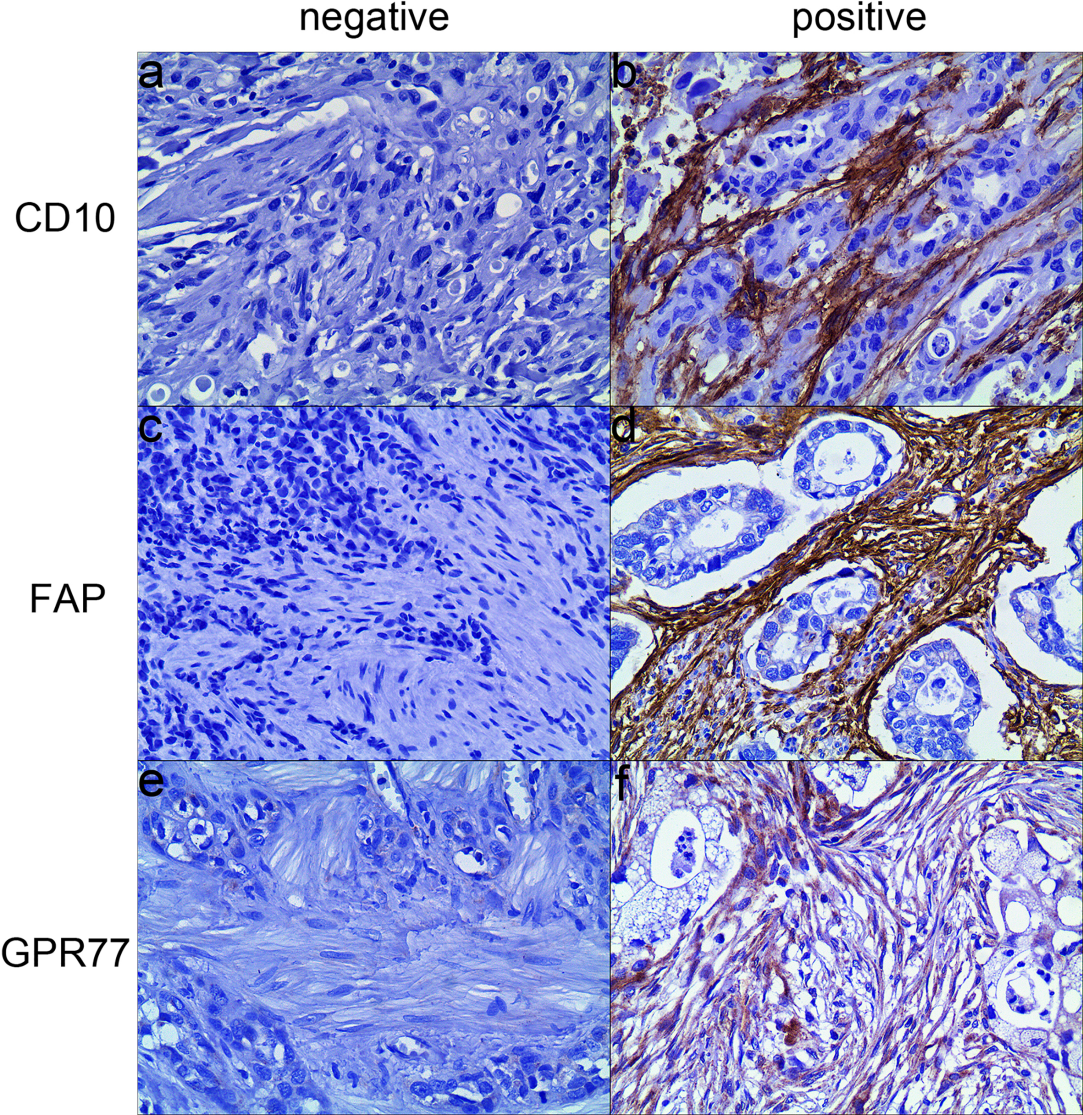
Representative examples of three biomarkers expression by immunohistochemistry (200×). **(A)** CD10 was negatively expressed in CAFs; **(B)** CD10 was positively expressed in the cytoplasm and membrane of CAFs; **(C)** FAP was negatively expressed in CAFs; **(D)** FAP was positively expressed in the cytoplasm of CAFs; **(E)** GRP77 was negatively expressed in CAFs; **(F)** GPR77 was positively expressed in the cytoplasm and membrane of CAFs.

Among 171 specimens before treatment, in CAFs, 26 (15.2%) cases were positive for CD10, 34 cases (19.9%) were positive for FAP, and 15 cases (8.8%) were positive for GPR77. Examples of expression of biomarkers were shown in **Figure 2**.

The expressions of biomarkers after treatment (post-) and before treatment (pre-) of all 171 patients are shown in **Table 1** and **Supplementary Table 1**, respectively. For biomarkers after treatment, high expressions of all biomarkers were related to worse pathological response (all p<0.005) (**Table 1**). However, before treatment, only high expression of pre-CD10 was related to worse pathological response (p=0.030), while pre-FAP (p=0.067) and pre-GPR77 (p=0.233) were not (**Supplementary Table 1**). The details of relationships between biomarkers and the TRG were shown in **Supplementary Table 2**.

**Table 1:** Correlation between biomarkers after treatment and clinicopathological characteristics.

	CD10		P-value	FAP		P-value	GPR77		P-value	No. (%)
	-	+		-	+		-	+	
Gender			0.716			0.140			0.221	
Male	104	24		73	55		110	18		128 (74.9)
Female	36	7		30	13		40	3		43 (25.1)
Age (yr)			0.228			0.793			0.417	
<65	113	22		82	53		117	18		135 (78.9)
≥65	27	9		21	15		33	3		36 (21.1)
Tumor location			0.339			0.725			0.255	
Lower third	74	15		51	38		74	15		89 (52.0)
Middle third	35	6		27	14		39	2		41 (24.0)
UGEJ	22	9		18	13		28	3		31 (18.1)
Diffuse	9	1		7	3		9	1		10 (5.8)
Tumor size (cm)			**0.006**			**0.002**			0.152	
<5	60	5		49	16		60	5		65 (38.0)
≥5	80	26		54	52		90	16		106 (62.0)
ypT			**0.047**			**<0.001**			0.106	
0	7	1		8	0		8	0		8 (4.7)
1-2	30	1		28	3		30	1		31 (18.1)
3-4	103	29		67	65		112	20		132 (77.2)
ypN			**0.034**			**0.034**			0.118	
0	57	6		49	14		60	3		63 (36.8)
1	26	5		20	11		26	5		31 (18.1)
2	19	10		14	15		23	6		29 (17.0)
3	38	10		20	28		41	7		48 (28.1)
ypTNM			**0.003**			**<0.001**			**0.014**	
II	30	2		30	2		31	1		32 (18.7)
II	40	3		29	14		41	2		43 (25.1)
III	70	26		44	52		78	18		96 (56.1)
Histological type			0.255			0.961			0.333	
Adenocarcinoma	84	22		64	42		95	11		106 (62.0)
Poorly cohesive carcinoma	56	9		39	26		55	10		65 (38.0)
Lauren classification			**0.006**			0.150			0.618	
Intestinal	66	23		49	40		77	12		89 (52.0)
Diffuse or Mixed	74	8		54	28		73	9		82 (48.0)
Grade of differentiation			0.116			**0.031**			0.064	
Well	31	3		26	8		33	1		34 (19.9)
Moderate or Poor	109	28		77	60		117	20		137 (80.1)
Vascular or lymphatic invasion			0.327			**0.016**			0.075	
No	106	26		86	46		119	13		132 (77.2)
Yes	34	5		17	22		31	8		39 (22.8)
Nervous invasion			0.219			**0.029**			0.367	
No	108	27		87	48		120	15		135 (78.9)
Yes	32	4		16	20		30	6		36 (21.1)
Adjuvant treatment			**0.037**			0.320			0.693	
No	13	7		10	10		17	3		20 (11.7)
Yes	127	24		93	58		133	18		151 (88.3)
Mandard TRG			**<0.001**			**<0.001**			**0.003**	
1-2	57	1		55	3		57	1		58 (33.9)
3-5	83	30		48	65		93	20		113 (66.1)

UGEJ, upper third and gastroesophageal junction; TRG, tumor regression grade. Bold values means significant.

### Relationships between biomarkers and other clinicopathological characteristics

The relationships between biomarkers after treatment and other clinicopathological characteristics are shown in **Table 1**. Post-CD10 and post-FAP were related to tumor size (p=0.006, p=0.002, respectively), ypT (p=0.047, p<0.001, respectively), and ypN (p=0.034, p=0.034, respectively). Post-CD10, post-FAP and post-GPR77 were related to TNM stage (p=0.003, p<0.001, p=0.014, respectively).

The relationships between biomarkers before treatment and other clinicopathological characteristics are shown in **Supplementary Table 1**. Pre-CD10 was related to tumor size (p=0.032), while pre-FAP was not (p=0.413). None of biomarkers before treatment were related to ypT and ypN. Only pre-GPR77 was related to ypTNM (p=0.014).

### Predictive value of biomarkers to pathological response

In univariable analysis for pathological response, for biomarkers after treatment, post-CD10 (odds ratio [OR], 20.602; p=0.003), post-FAP (OR, 24.826; p<0.001), and post-GPR77 (OR, 12.258; p=0.016) were predictors to pathological response. However, for biomarkers before treatment, only pre-CD10 (OR, 3.264; p=0.038) were related to pathological reaction (**Table 2**).

**Table 2:** Univariable analysis for pathological response.

Variable	OR (95%CI)	P
Gender (Female)	0.721 (0.353, 1.474)	0.370
Age (≥65yr)	0.760 (0.355, 1.626)	0.479
Tumor location		0.444
UGEJ	1	
Middle third	0.892 (0.344, 2.313)	0.814
Lower third	1.450 (0.618, 3.401)	0.393
Diffuse	2.526 (0.457, 13.964)	0.288
Tumor size (≥5cm)	6.052 (3.052, 12.101)	**< 0.001**
ypN		**< 0.001**
0	1	
1	1.429 (0.600, 3.404)	0.420
2	6.452 (2.012, 20.690)	**0.002**
3	4.473 (1.861, 10.753)	**< 0.001**
ypTNM (III)	6.364 (3.147, 12.869)	**< 0.001**
Histological type	2.275 (1.135, 4.559)	**0.020**
Lauren classification	1.663 (0.874, 3.162)	0.121
Grade of differentiation	4.336 (1.986, 9.599)	**< 0.001**
Vascular or lymphatic invasion	3.575 (1.400, 9.127)	**0.008**
Nervous invasion	4.007 (1.466, 10.956)	**0.007**
Adjuvant therapy	0.616 (0.212, 1.789)	0.374
Post-treatment		
CD10 (+)	20.602 (2.731, 155.415)	**0.003**
FAP (+)	24.826 (7.326, 84.128)	**< 0.001**
GPR77 (+)	12.258 (1.601, 93.825)	**0.016**
Pre-treatment		
CD10 (+)	3.264 (1.068, 9.976)	**0.038**
FAP (+)	2.287 (0.929, 5.629)	0.072
GPR77 (+)	2.178 (0.589, 8.049)	0.243

UGEJ, upper third and gastroesophageal junction. Bold values means significant.

For other clinicopathological factors, tumor size (OR, 6.502; p<0.001), ypN (OR, 4.473; p<0.001), ypTNM (OR, 6.364; p<0.001), histological type (OR, 2.275; p=0.020), grade of differentiation (OR, 4.436; p<0.001), vascular or lymphatic invasion (OR, 3.575; p=0.008), nervous invasion (OR, 4.007; p=0.007) were related to pathological response (**Table 2**).

Four groups of biomarkers were included in multivariable analyses for pathological response respectively, with other statistically significant factors. Post-FAP (OR, 12.805; p<0.001) and pre-FAP (OR, 5.672; p=0.009) were independent predictors for pathological response, respectively, while CD10 and GPR77 were not (**Table 3**).

**Table 3:** Multivariable analysis for pathological response.

Variable	OR (95%CI)	P
Post-treatment		
CD10 (+)	8.044 (0.603, 107.347)	0.115
FAP (+)	12.805 (3.129, 52.406)	**< 0.001**
GPR77 (+)	1.694 (0.149, 19.232)	0.671
Pre-treatment		
CD10 (+)	1.411 (0.364, 5.473)	0.619
FAP (+)	5.672 (1.529, 21.046)	**0.009**
GPR77 (+)	0.920 (0.177, 4.769)	0.921

All factors with p < 0.05 in univariable analysis except ypTNM were included. Biomarkers after treatment (post-) and before treatment (pre-) were included in multivariable analysis respectively. Bold values means significant.

### Prognostic value of biomarkers

**Figure 3:**
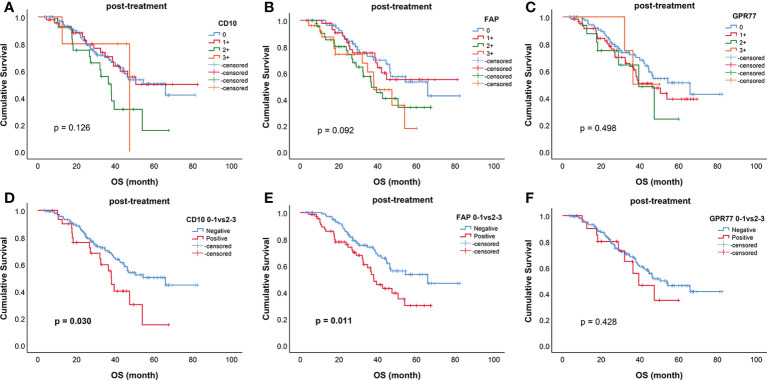
Kaplan–Meier curves for overall survival (OS) of biomarkers after treatment. Survival curves for **(A)** CD10; **(B)** FAP; **(C)** GPR77; **(D)** CD10 (0-1 vs 2-3); **(E)** FAP (0-1 vs 2-3); **(F)** GPR77 (0-1 vs 2-3). CD10 (0-1 vs 2-3) (p=0.030) and FAP (0-1 vs 2-3) (p=0.011) were significantly related to OS.

The survival curves of all biomarkers after treatment were shown in **Figure 3**. When divided into two groups, post-CD10 (p=0.030), post-FAP (p=0.011) were related to OS (**Figure 3**). However, for biomarkers before treatment, only pre-FAP was related to OS (p=0.024) (**Supplementary Figure 2**).

In univariable analysis for OS, post-CD10 (hazard ratio [HR], 1.832; p=0.032), post-FAP (HR, 1.843; p=0.013) were related to the prognosis. None of biomarkers before treatment were related to the prognosis (all p > 0.05) (**Table 4**).

**Table 4:** Univariable analysis for overall survival.

Variable	HR (95%CI)	P
Gender (Female)	1.474 (0.852, 2.550)	0.165
Age (≥65yr)	1.402 (0.816, 2.406)	0.221
Tumor location		**0.008**
UGEJ	1	
Middle third	2.227 (0.936, 5.301)	0.070
Lower third	1.405 (0.619, 3.189)	0.417
Diffuse	4.539 (1.637, 12.581)	**0.004**
Tumor size (≥5cm)	3.092 (1.684, 5.680)	**< 0.001**
ypT		**0.001**
0	1	
1-2	0.810 (0.084, 7.791)	0.885
3-4	6.235 (0.864, 45.012)	0.070
ypN		**< 0.001**
0	1	
1	3.280 (1.456, 7.390)	**0.004**
2	2.928 (1.264, 6.783)	**0.012**
3	8.676 (4.192, 17.956)	**< 0.001**
ypTNM (III)	4.442 (2.456, 8.036)	**< 0.001**
Histological type	1.167 (0.716, 1.904)	0.535
Lauren classification	1.925 (1.178, 3.144)	**0.009**
Grade of differentiation	2.733 (1.303, 5.731)	**0.008**
Vascular or lymphatic invasion	1.901 (1.126, 3.210)	**0.016**
Nervous invasion	1.256 (0.716, 2.206)	0.427
Mandard TRG (3–5)	2.861 (1.557, 5.260)	**0.001**
Post-treatment		
CD10 (+)	1.832 (1.053, 3.189)	**0.032**
FAP (+)	1.843 (1.139, 2.983)	**0.013**
GPR77 (+)	1.329 (0.657, 2.690)	0.429
Pre-treatment		
CD10 (+)	1.293 (0.676, 2.475)	0.437
FAP (+)	0.698 (0.373, 1.305)	0.260
GPR77 (+)	0.977 (0.422, 2.265)	0.957

UGEJ, upper third and gastroesophageal junction; TRG, tumor regression grade. Bold values means significant.

In multivariable analysis, four groups of biomarkers were included respectively. None of biomarkers were independent factors for OS (all p > 0.05) (**Table 5**).

**Table 5:** Multivariable analysis for overall survival.

Variable	HR (95%CI)	P
Post-treatment		
CD10 (+)	1.929 (0.899, 4.140)	0.092
FAP (+)	0.755 (0.371, 1.536)	0.439
GPR77 (+)	1.007 (0.440, 2.302)	0.988
Pre-treatment		
CD10 (+)	1.453 (0.725, 2.910)	0.292
FAP (+)	0.604 (0.295, 1.237)	0.168
GPR77 (+)	1.099 (0.438, 2.758)	0.840

All factors with p < 0.05 in univariable analysis except ypTNM were included. Biomarkers after treatment (post-) and before treatment (pre-) were included in multivariable analysis respectively.

## Discussion

In this study, we investigated the relationships between expressions of CD10, FAP, GPR77 in CAFs and clinicopathological characteristics; and explored the predictive values of these biomarkers before and after treatment for pathological response and OS. We found that high expressions of post-CD10, post-FAP, and post-GPR77 predicted a worse pathological response, and post-FAP was independent predictive factor to pathological response. These results are consistent with other studies, in which FAP was proved to be related to drug chemoresistance (15, 24, 25). The mechanisms of FAP increasing drug resistance are various, such as promoting immunosuppression (13), interacting with membrane proteins (14) and producing chemokine (15). Drugs targeting FAP have shown great effect *in vitro* experiments (26, 27), but *in vivo*, these drugs did not show satisfactory effectiveness (28). CD10 has also been proved to promote cancer formation and chemoresistance in breast cancer (8), colorectal cancer (29) and malignant melanoma (20), involving mechanisms such as providing a survival niche for cancer stem cells (8) and promoting epithelial–mesenchymal transition (29). For GPR77, one study suggested it was related to cancer formation and chemoresistance (8).

In our study, post-CD10 and post-FAP were related to prognosis. This result is in line with other studies. High expression of CD10 has been proved to be related to a poor prognosis in breast cancer (22), malignant melanoma (20) and esophageal carcinoma (30). Nevertheless, CD10 did not show similar predictive value in papillary thyroid carcinoma (31). In gastric cancer, few articles verified this conclusion, especially based on patients who underwent NCT. High expression of FAP has been proved to be related to a poor prognosis in pancreatic ductal adenocarcinoma (32), colorectal cancer (17) and gastric cancer (16, 18, 33), but none of their patients underwent NCT. In Wen et al. (16) study, high FAP expression was an independent prognostic factor of poor survival in GC patients, but in our study, none of biomarkers were independent prognostic factors. This might be attributed to the influence of NCT, or the collinearity among biomarkers. In our study, GPR77 did not show prognostic predictive value, but Su et al. (8) suggested GPR77 was related to chemoresistance in breast cancer. Therefore, more evidences are needed to verify these results.

It is worthy to mention that in our study, predictive values of biomarkers after treatment were more significant than those before treatment. This might because the roles of biomarkers changed due to NCT, or because the preoperative biopsy specimens were not enough to show the roles of biomarkers completely. Nevertheless, these results suggested that biomarkers after treatment had better predictive values for efficacy of NCT and prognosis.

In addition, we found that after treatment, high expressions of CD10, FAP and GPR77 were related to T stage and TNM stage, which is in accordance with other articles (18, 33, 34). Hu et al. (18) suggested FAP was related to histological type, while FAP did not show similar result in our study. This difference might due to the influence of NCT.

We explored the relationships between these biomarkers of CAFs and chemoresistance clinically, but more researches on mechanism are needed. In addition, in our study, CD10, and FAP before treatment are related to pathological reaction, which means these biomarkers have the potential to predict the efficacy of NCT and could be helpful to further clinical decision-making.

This study has several limitations. It was a retrospective study from a single institution and the sample size was not large, which might cause bias. The relationships between biomarkers and pathological response and prognosis were investigated clinically, but we have not yet revealed the mechanism that lead to these results. When taking pre-treatment specimens, due to the limitation of the amount of pre-treatment specimens, false-negative results might occur, which might be the reason that some pre-treatment markers did not show prognostic predictive value. Because of correlations of expression locations of biomarkers, collinearity might be the reason why some biomarkers lost predictive values in multivariable analysis. Nevertheless, we concentrated on a specific group of patients and verified the clinical values of CAFs biomarkers, including CD10, FAP and GPR77 in locally advanced gastric cancer patients who underwent NCT, and explored the association of these biomarkers with drug resistance and prognosis. These results could be helpful to clinical decision making and could provide evidence for future researches.

In conclusion, the expressions of CD10, FAP and GPR77 in CAFs were related to drug resistance and overall survival, and they could be used as predictors for pathological reaction and prognosis in locally advanced gastric cancer patients.

## Data availability statement

The original contributions presented in the study are included in the article/**Supplementary Material**. Further inquiries can be directed to the corresponding authors.

## Ethics statement

All experimental protocols were approved by the Institutional Review Board (IRB) at Liaoning Cancer Hospital and Institute (Cancer Hospital of China Medical University). All methods were carried out in accordance with relevant guidelines and regulations. The patients/participants provided their written informed consent to participate in this study. Written informed consent was obtained from the individual(s) for the publication of any potentially identifiable images or data included in this article.

## Author contributions

YT and ZZ performed the majority of experiments and analyzed the data and drafted the manuscript. JZ reviewed and revised the manuscript. WW assisted in collecting and analyzing the data. YZ supervised the study and provided critical revision of the manuscript. All authors contributed to the article and approved the submitted version.

## Funding

This work was supported by grants from the Natural Science Foundation of Liaoning Province (2020-ZLLH-45), Shenyang High-level Innovative Talents Program (RC190447) and Liaoning Cancer Hospital & Institute- Dalian University of Technology “Medical-industrial interdisciplinary research fund” (LD202021).

## Conflict of interest

The authors declare that the research was conducted in the absence of any commercial or financial relationships that could be construed as a potential conflict of interest.

## Publisher’s note

All claims expressed in this article are solely those of the authors and do not necessarily represent those of their affiliated organizations, or those of the publisher, the editors and the reviewers. Any product that may be evaluated in this article, or claim that may be made by its manufacturer, is not guaranteed or endorsed by the publisher.

## Abbreviations

NCT, neoadjuvant therapy; GC, gastric cancer; TRG, tumor regression grade; CAFs, cancer-associated fibroblasts; ECM, extracellular matrix; FAP, Fibroblast-activation protein; GPR77, G-protein-coupled receptor 77; AJCC, American Joint Committee on Cancer; OS, overall survival; ypTNM, post-neoadjuvant therapy stage.
