# Supplementary material for: High expressions of CD10, FAP and GPR77 in CAFs are associated with chemoresistance and worse prognosis in gastric cancer
Source: Front Oncol. 2022 Oct 28;12:984817. doi: 10.3389/fonc.2022.984817 (PMC9650088; doi:10.3389/fonc.2022.984817)
Supplement: Supplementary file 1 [file Image_1.pdf]

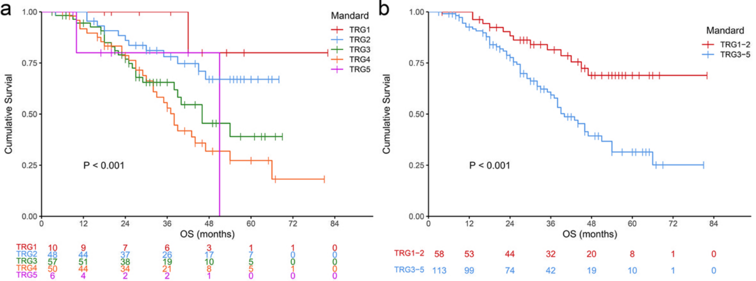

Supplementary Figure 1. Kaplan–Meier curves for overall survival (OS) of Mandard TRG (a-b): (a) 5-grade TRG. (b) pathological response (TRG 1-2 vs 3-5). Both TRG ($p<0.001$) and TRG (1-2 vs 3-5) ($p<0.001$) were significantly related to OS.
