# Supplementary material for: High expressions of CD10, FAP and GPR77 in CAFs are associated with chemoresistance and worse prognosis in gastric cancer
Source: Front Oncol. 2022 Oct 28;12:984817. doi: 10.3389/fonc.2022.984817 (PMC9650088; doi:10.3389/fonc.2022.984817)
Supplement: Supplementary file 2 [file Image_2.pdf]

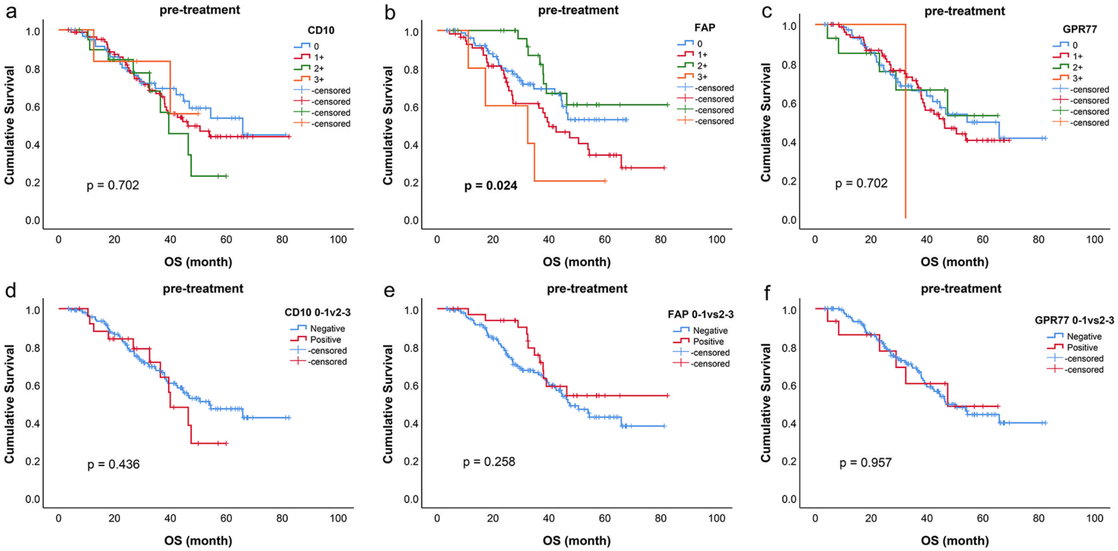

Supplementary Figure 2. Kaplan–Meier curves for overall survival (OS) of biomarkers before treatment. Survival curves for (a) CD10; (b) FAP; (c) GPR77; (d) CD10 (0-1 vs 2-3); (e) FAP (0-1 vs 2-3); (f) GPR77 (0-1 vs 2-3). FAP was significantly related to OS (p=0.024).
