# Supplementary material for: High expressions of CD10, FAP and GPR77 in CAFs are associated with chemoresistance and worse prognosis in gastric cancer
Source: Front Oncol. 2022 Oct 28;12:984817. doi: 10.3389/fonc.2022.984817 (PMC9650088; doi:10.3389/fonc.2022.984817)
Supplement: Supplementary file 3 [file Table_1.docx]

| Supplementary Table 1 Correlation between biomarkers before treatment and clinicopathological characteristics | | | | | | | | | | |
| --- | --- | --- | --- | --- | --- | --- | --- | --- | --- | --- |
|  | CD10 | | P-value | FAP | | P-value | GPR77 | | P-value | No. (%) |
|  | - | + |  | - | + |  | - | + |  |  |
| Gender |  |  | 0.450 |  |  | 0.494 |  |  | **0.019** |  |
| Male | 107 | 21 |  | 101 | 27 |  | 113 | 15 |  | 128 (74.9) |
| Female | 38 | 5 |  | 36 | 7 |  | 43 | 0 |  | 43 (25.1) |
| Age |  |  | 0.441 |  |  | 0.692 |  |  | 0.443 |  |
| ＜65 | 113 | 22 |  | 109 | 26 |  | 122 | 13 |  | 135 (78.9) |
| ≥65 | 32 | 4 |  | 28 | 8 |  | 34 | 2 |  | 36 (21.1) |
| Tumor location |  |  | 0.372 |  |  | 0.073 |  |  | 0.496 |  |
| Lower third | 73 | 16 |  | 66 | 23 |  | 81 | 8 |  | 89 (52.0) |
| Middle third | 38 | 3 |  | 38 | 3 |  | 39 | 2 |  | 41 (24.0) |
| UGEJ | 25 | 6 |  | 26 | 5 |  | 28 | 3 |  | 31 (18.1) |
| Diffuse | 9 | 1 |  | 7 | 3 |  | 8 | 2 |  | 10 (5.8) |
| Tumor size (cm) |  |  | **0.032** |  |  | 0.413 |  |  | 0.696 |  |
| <5 | 60 | 5 |  | 50 | 15 |  | 60 | 5 |  | 65 (38.0) |
| ≥5 | 85 | 21 |  | 87 | 19 |  | 96 | 10 |  | 106 (62.0) |
| ypT |  |  | 0.411 |  |  | 0.155 |  |  | 0.467 |  |
| 0 | 8 | 0 |  | 5 | 3 |  | 7 | 1 |  | 8 (4.7) |
| 1-2 | 27 | 4 |  | 28 | 3 |  | 30 | 1 |  | 31 (18.1) |
| 3-4 | 110 | 22 |  | 104 | 28 |  | 119 | 13 |  | 132 (77.2) |
| ypN |  |  | 0.738 |  |  | 0.662 |  |  | 0.420 |  |
| 0 | 53 | 10 |  | 48 | 15 |  | 59 | 4 |  | 63 (36.8) |
| 1 | 28 | 3 |  | 26 | 5 |  | 29 | 2 |  | 31 (18.1) |
| 2 | 25 | 4 |  | 25 | 4 |  | 27 | 2 |  | 29 (17.0) |
| 3 | 39 | 9 |  | 38 | 10 |  | 41 | 7 |  | 48 (28.1) |
| ypTNM |  |  | 0.506 |  |  | 0.972 |  |  | **0.014** |  |
| Ⅰ | 29 | 3 |  | 26 | 6 |  | 30 | 2 |  | 32 (18.7) |
| Ⅱ | 37 | 6 |  | 34 | 9 |  | 40 | 3 |  | 43 (25.1) |
| Ⅲ | 79 | 17 |  | 77 | 19 |  | 86 | 10 |  | 96 (56.1) |
| Histological type |  |  | 0.959 |  |  | 0.248 |  |  | 0.343 |  |
| Adenocarcinoma | 90 | 16 |  | 82 | 24 |  | 95 | 11 |  | 106 (62.0) |
| Poorly cohesive carcinoma | 55 | 10 |  | 55 | 10 |  | 61 | 4 |  | 65 (38.0) |
| Lauren classification |  |  | 0.293 |  |  | **0.005** |  |  | 0.235 |  |
| Intestinal | 73 | 16 |  | 64 | 25 |  | 79 | 10 |  | 89 (52.0) |
| Diffuse or Mixed | 72 | 10 |  | 73 | 9 |  | 77 | 5 |  | 82 (48.0) |
| Grade of differentiation |  |  | 0.091 |  |  | **0.012** |  |  | 0.991 |  |
| Well | 32 | 2 |  | 22 | 12 |  | 31 | 3 |  | 34 (19.9) |
| Moderate or Poor | 113 | 24 |  | 115 | 22 |  | 125 | 12 |  | 137 (80.1) |
| Vascular or lymphatic invasion |  |  | 0.637 |  |  | 0.305 |  |  | 0.309 |  |
| No | 111 | 21 |  | 108 | 24 |  | 122 | 10 |  | 132 (77.2) |
| Yes | 34 | 5 |  | 29 | 10 |  | 34 | 5 |  | 39 (22.8) |
| Nervous invasion |  |  | 0.187 |  |  | 0.692 |  |  | 0.222 |  |
| No | 117 | 18 |  | 109 | 26 |  | 125 | 10 |  | 135 (78.9) |
| Yes | 28 | 8 |  | 28 | 8 |  | 31 | 5 |  | 36 (21.1) |
| Adjuvant treatment |  |  | 0.525 |  |  | 0.989 |  |  | 0.836 |  |
| No | 16 | 4 |  | 16 | 4 |  | 18 | 2 |  | 20 (11.7) |
| Yes | 129 | 22 |  | 121 | 30 |  | 138 | 13 |  | 151 (88.3) |
| Mandard TRG |  |  | **0.030** |  |  | 0.067 |  |  | 0.233 |  |
| 1-2 | 54 | 4 |  | 51 | 7 |  | 55 | 3 |  | 58 (33.9) |
| 3-5 | 91 | 22 |  | 86 | 27 |  | 101 | 12 |  | 113 (66.1) |
| Note: UGEJ, upper third and gastroesophageal junction; TRG, tumor regression grade | | | | | | | | | | |
