# Supplementary material for: High expressions of CD10, FAP and GPR77 in CAFs are associated with chemoresistance and worse prognosis in gastric cancer
Source: Front Oncol. 2022 Oct 28;12:984817. doi: 10.3389/fonc.2022.984817 (PMC9650088; doi:10.3389/fonc.2022.984817)
Supplement: Supplementary file 4 [file Table_2.docx]

| Supplementary Table 2 Association between biomarkers in CAFs and Mandard TRG | | | | | |
| --- | --- | --- | --- | --- | --- |
| Variable | TRG 1-2 (n=58) (%) | TRG 3 (n=57) (%) | TRG 4-5 (n=56) (%) | P | No. (%) |
| Post-treatment |  |  |  |  |  |
| CD10 |  |  |  | **<0.001** |  |
| 0 | 47 (27.5) | 32 (18.7) | 15 (8.8) |  | 94 (55.0) |
| 1+ | 10 (5.8) | 19 (11.1) | 17 (9.9) |  | 46 (26.9) |
| 2+ | 1 (0.6) | 6 (3.5) | 19 (11.1) |  | 26 (15.2) |
| 3+ | 0 (0.0) | 0 (0.0) | 5 (2.9) |  | 5 (2.9) |
| FAP |  |  |  | **<0.001** |  |
| 0 | 36 (21.1) | 16 (9.4) | 7 (4.1) |  | 59 (34.5) |
| 1+ | 19 (11.1) | 18 (10.5) | 7 (4.1) |  | 44 (25.7) |
| 2+ | 3 (1.8) | 19 (11.1) | 21 (12.3) |  | 43 (25.1) |
| 3+ | 0 (0.0) | 4 (2.3) | 21 (12.3) |  | 25 (14.6) |
| GPR77 |  |  |  | **<0.001** |  |
| 0 | 50 (29.2) | 27 (15.8) | 12 (7.0) |  | 89 (52.0) |
| 1+ | 7 (4.1) | 23 (13.5) | 31 (18.1) |  | 61 (35.7) |
| 2+ | 1 (0.6) | 5 (2.9) | 11 (6.4) |  | 17 (9.9) |
| 3+ | 0 (0.0) | 2 (1.2) | 2 (1.2) |  | 4 (2.3) |
| Pre-treatment |  |  |  |  |  |
| CD10 |  |  |  | **<0.001** |  |
| 0 | 36 (21.1) | 16 (9.4) | 10 (5.8) |  | 62 (36.3) |
| 1+ | 18 (10.5) | 31 (18.1) | 34 (19.9) |  | 83 (48.5) |
| 2+ | 4 (2.3) | 7 (4.1) | 9 (5.3) |  | 20 (11.7) |
| 3+ | 0 (0.0) | 3 (1.8) | 3 (1.8) |  | 6 (3.5) |
| FAP |  |  |  | **<0.001** |  |
| 0 | 44 (25.7) | 24 (14.0) | 13 (7.6) |  | 81 (47.4) |
| 1+ | 7 (4.1) | 19 (11.1) | 30 (17.5) |  | 56 (32.7) |
| 2+ | 7 (4.1) | 13 (7.6) | 9 (5.3) |  | 29 (17.0) |
| 3+ | 0 (0.0) | 1 (0.6) | 4 (2.3) |  | 5 (2.9) |
| GPR77 |  |  |  | **<0.001** |  |
| 0 | 44 (25.7) | 21 (12.3) | 12 (7.0) |  | 77 (45.0) |
| 1+ | 11 (6.4) | 30 (17.5) | 38 (22.2) |  | 79 (46.2) |
| 2+ | 3 (1.8) | 6 (3.5) | 5 (2.9) |  | 14 (8.2) |
| 3+ | 0 (0.0) | 0 (0.0) | 1 (0.6) |  | 1 (0.6) |

Note: TRG, tumor regression grade
